# Endometrial carcinoma in a gravid uterus: a case report and literature review

**DOI:** 10.1186/s12884-019-2489-y

**Published:** 2019-11-20

**Authors:** Mayu Shiomi, Shinya Matsuzaki, Eiji Kobayashi, Takeya Hara, Satoshi Nakagawa, Tsuyoshi Takiuchi, Kazuya Mimura, Yutaka Ueda, Takuji Tomimatsu, Tadashi Kimura

**Affiliations:** Department of Obstetrics and Gynecology, Osaka University Graduate School of Medicine, 2-2 Yamadaoka, Suita, Osaka, 565-0871 Japan

**Keywords:** Placenta accreta spectrum, Placenta previa, Pregnancy, Endometrioid carcinoma endometrial carcinoma, Endometrial cancer

## Abstract

**Background:**

Endometrial carcinoma (EC) is rarely diagnosed during pregnancy. Therefore, the histopathological findings, clinical course, and gross appearance of the resected uterus during pregnancy are not well known. We present a case of EC diagnosed during pregnancy. In addition, we reviewed the literature dating from January 1995 to March 2019 for cases of EC diagnosed during pregnancy and within 15 months after pregnancy, and we discussed this topic to improve the understanding of this rare condition.

**Case presentation:**

A 35-year-old woman underwent an urgent cesarean delivery in gestational week 35 due to antepartum bleeding caused by placenta previa. Hysterectomy was performed with the diagnosis of placenta accreta spectrum (PAS). Remarkably, the postoperative gross and histopathological examinations revealed an endometrioid adenocarcinoma (grade 1). The histopathological findings revealed a pattern similar to that of EC not related with pregnancy. Immunohistochemistry revealed an overexpression of the estrogen and progesterone receptors; however, the p53 expression was negative. We performed laparoscopic bilateral salpingo-oophorectomy and pelvic lymphadenectomy 102 days after the cesarean hysterectomy, and confirmed surgical stage IA without metastases. Our patient has had no recurrence in 4 years after the cesarean delivery.

An electronic search of the literature revealed 25 cases of EC (including our case) diagnosed during or after pregnancy. Sixteen of the 25 patients were diagnosed after abortions in the first trimester, 9 were diagnosed within 14 months of childbirth, and our case was the first with diagnosis from a surgical specimen of peripartum hysterectomy due to the PAS. In 23 of the 25 cases endometrioid adenocarcinoma grade 1 to 2 was found, and it seemed to have a good prognosis.

**Conclusion:**

The present findings suggest that careful examination of a resected uterus is essential, even when surgery is performed for an obstetric indication. Our case is an extremely rare case of EC during pregnancy; the histopathological pattern was similar to that of typical EC, and no recurrence was noted. The high levels of estrogen and progesterone during pregnancy did not seem to promote tumor progression in our case.

This study presents a case of endometrioid carcinoma diagnosed during pregnancy. We performed literature review and discussed this topic. We have discussed the effects of pregnancy on endometrioid carcinoma in a previous study. Our present study found the points listed below.

1. Although endometrial carcinoma during pregnancy is extremely rare, careful observation of the resected uterus is needed to avoid a missed diagnosis.
2. In our case, histopathological and immunohistochemical findings were consistent with endometrioid adenocarcinoma grade 1. The patient has been disease-free for about 4 years after cesarean hysterectomy. The high levels of estrogen and progesterone during pregnancy did not seem to promote tumor progression in our case.
3. Although high levels of estrogen (which has a promoting effect on endometrioid carcinoma) and progesterone (which has an anti-tumor effect on endometrioid carcinoma) were observed, most authors reported that the endometrioid carcinoma associated with pregnancy had a good prognosis with minimal myometrial invasion.

## Background

Endometrial carcinoma (EC) is the fourth most common cancer in women in high-income countries; however, EC commonly occurs in peri- or postmenopausal women, and only 5% of women are diagnosed with adenocarcinoma before the age of 40 years [1, 2]. Therefore, the coexistence of EC and pregnancy is rare. Moreover, EC is rarely detected during pregnancy or within a year postpartum because the tumor can disrupt the pregnancy. Although a previous study had already reviewed the latest 35 reports on EC coexisting with pregnancy during the last 80 years [3], the outcome of EC associated with pregnancy and the effect of pregnancy on EC is not well known. Previous literature review also showed that there have been no reports of diagnosing EC during pregnancy in the surgical specimen of cesarean hysterectomy. Therefore, we report a case of EC diagnosed in a postoperative histopathological examination after total hysterectomy for placenta accreta spectrum (PAS), and we additionally present the results of a literature review on this matter.

## Case presentation

**Fig. 1:**
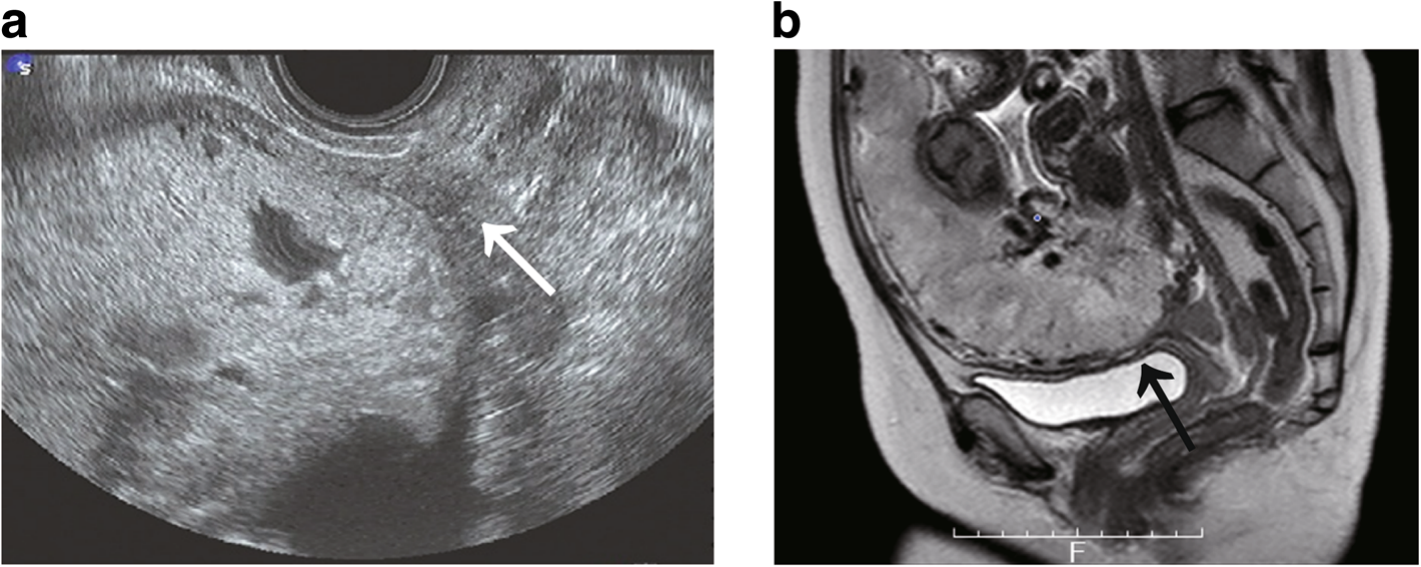
Images for assessment of placenta accreta spectrum. **a** Transvaginal ultrasonography shows total placenta previa with one lacuna. **b** Magnetic resonance imaging (MRI) at gestational week 31 revealed total placenta previa, and the placenta was located mainly on the anterior side. Although intraplacental T2 dark band, uterine bulging, and heterogeneous placenta were not observed, we found myometrial thinning of the anterior wall and loss of myometrium between the placenta and bladder wall. The black arrow indicates loss of uterine myometrium between the placenta and bladder wall. Based on these findings, we suspected placenta accreta spectrum. No abnormal finding was observed in the fetus

A 35-year-old woman (gravida 2, para 1) was referred to our hospital due to placenta previa at gestational week 31. Her medical history was unremarkable, and her previous pregnancy was an uncomplicated, normal vaginal delivery at gestational week 38. Her current pregnancy was uncomplicated except for the placenta previa. She denied abnormal genital bleeding before the current pregnancy. Cervical cytology performed during early pregnancy was negative for intraepithelial lesions. Vaginal ultrasonography revealed total placenta previa and one lacuna (Fig. 1a). Magnetic resonance imaging (MRI) at gestational week 31 revealed total placenta previa and loss of the myometrium between the placenta and bladder wall (Fig. 1b). Other MRI findings of PAS such as uterine bulging, heterogenous placenta, and T2 dark band were not observed. Based on these findings, we suspected PAS, and an emergency cesarean delivery was performed owing to antepartum bleeding (approximately 100 mL) at gestational week 35. An abdominal midline incision was made, and a healthy male infant weighing 2274 g (− 0.42 SD) was delivered with Apgar scores of 8 and 9, at 1 and 5 min, respectively. The placenta was not delivered within 30 min after fetal delivery, thus requiring hysterectomy for PAS. Estimated blood loss was 1000 mL. The postoperative course was uneventful, and the patient and baby were discharged on the 8th postoperative day.

**Fig. 2:**
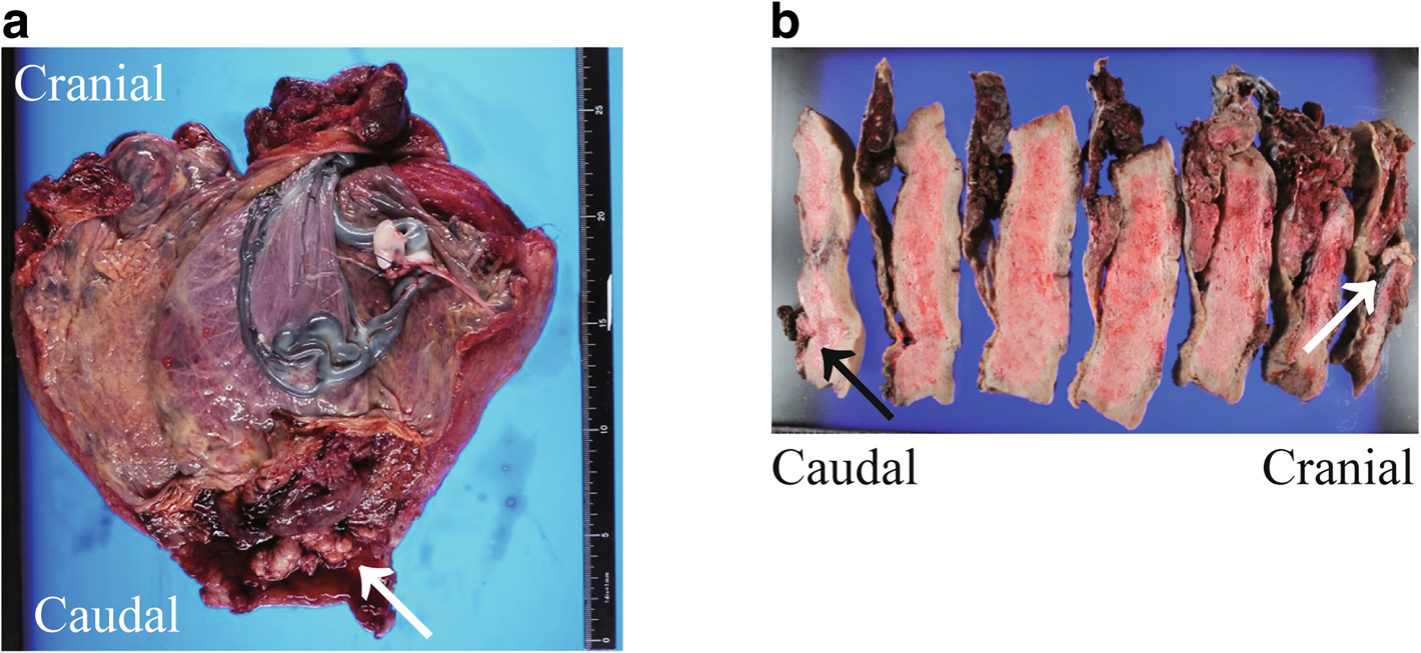
Macroscopic findings in the surgical specimen. **a** The image shows gross findings in the uterus, which was resected due to placenta accreta spectrum. The white arrow indicates a white tumor measuring 3 cm in diameter, involving the lower uterine segment, which was diagnosed as endometrial carcinoma by histopathological analysis. The tumor involving the uterine fundus is not identifiable because it is covered by the placenta. **b** The image shows a longitudinal section of the uterus, which was divided into 7 sections. After the placenta was removed, a white tumor measuring 2 cm in diameter involving the uterine fundal segment was seen. The black arrow indicates the 3-cm diameter tumor which was endometrial carcinoma involving the lower uterine segment; the white arrow indicates the tumor involving the uterine fundus. Both tumors were soft and white, and the macroscopic findings were similar in both tumors

**Fig. 3:**
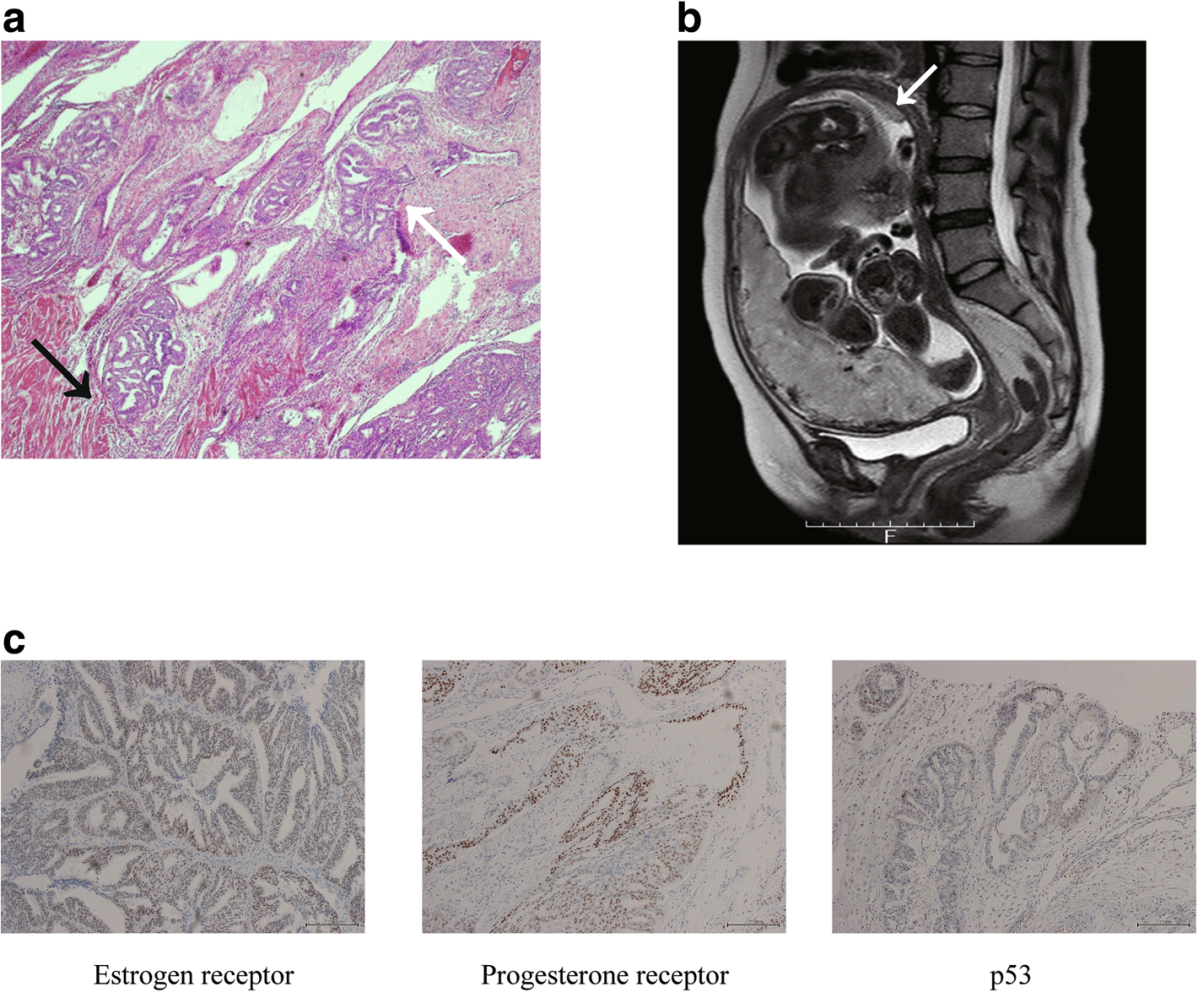
Postoperative analysis of histopathological findings, magnetic resonance imaging, and immunohistochemistry staining. **a** The image shows the histopathological findings in the resected uterine specimen. Well-differentiated adenocarcinoma with focal cribriform pattern, back-to-back structure without intervening stroma, and a papillary area are observed, and the glands have a smooth luminal contour. The tumor shows predominant glandular growth and a < 5% nonsquamous solid component; thus, the tumor was diagnosed as endometrial cancer grade 1. The tumor at the lower uterine segment shows slight myometrial invasion. The white arrow indicates the tumor in the uterine lower segment which shows invasion of the placenta decidua and uterine myometrium. The black arrow indicates < 50% myometrial invasion (hematoxylin and eosin stain, × 40.) **b** Immunohistochemistry analysis showed positive expression of estrogen and progesterone receptors, and negative expression of p53. (Magnification, × 40.) **c** Retrospectively reviewed magnetic resonance imaging (MRI) revealed endometrial carcinoma in the uterine fundus. A sagittal T2-weighted MR image shows endometrial carcinoma measuring 3 cm in diameter with signal intensity resembling that of the placenta. The white arrow indicates endometrial carcinoma involving the uterine fundus

Part of the chorion and placenta were adhered to the uterus (Fig. 2a). The resected uterus was divided to 7 specimens in order to perform macroscopic and histopathological analyses. The surgical specimen showed a white polyp measuring 2 cm, which parted from the uterine fundus and the lower uterine segment (Fig. 2b). Histopathological examination of the tumor involving the lower uterine segment revealed endometrioid adenocarcinoma (Grade 1), with < 50% myometrial invasion and positive expression of estrogen and progesterone receptors, in addition to PAS (Fig. 3a and b). Notably, the tumor involving the uterine fundus did not show myometrial invasion. Histopathological findings were similar in both tumors located in the uterine lower segment and uterine fundus. A retrospective review of the MRI images obtained during pregnancy revealed the tumor involving the uterine fundus, although involvement of the lower uterine segment was difficult to detect (Fig. 3c). We performed a laparoscopic bilateral salpingo-oophorectomy and pelvic lymphadenectomy 102 days after cesarean hysterectomy and confirmed the absence of metastases. The tumor was a stage IA lesion based on the International Federation of Gynecology and Obstetrics system. Follow-up performed 4 years after cesarean hysterectomy revealed no recurrence.

## Discussion and conclusion

Our case demonstrated the gross and histopathological findings, MRI findings, and clinical course of EC during pregnancy. To discuss this rare condition, we performed a literature review of cases of endometrioid carcinoma associated with pregnancy. We defined EC associated with pregnancy as diagnosed at delivery to within 15 months after pregnancy. We performed a search of PubMed, MEDLINE, and Scopus databases for the period between January 1995 and March 2019, using the following key words: “endometrial cancer”, “endometrial carcinoma”, “endometrioid cancer”, “endometrioid carcinoma”, “corpus cancer”, “pregnancy”, “abortion”, and “postpartum” in various combinations. We excluded non-English articles, discontinued journal and those published before 1995. We summarized the timing of diagnosis, outcome of EC, symptoms, diagnosis of histopathological examination, surgical stage (base on FIGO 2008) [4], and surgical treatment for EC. We also listed the authors’ opinions and discussions about the effect of pregnancy on the prognosis of EC.

A total of 18 studies with 25 cases of EC associated with pregnancy (including our case) have been reported [3, 5–20]; 9 cases were identified postpartum up to the 14-month; 16 cases were diagnosed at the time of D&C for first-trimester spontaneous or elective abortion. These results suggest that clinicians should consider EC after pregnancy even though abnormal bleeding is often observed after pregnancy, and EC associated with pregnancy is rare. Our literature review revealed that there were no previous reports of a diagnosis of EC based on an examination of the resected uterus following cesarean hysterectomy for PAS [3, 7]. Although our case is extremely rare, clinicians should check the macroscopic finding of the resected uterus carefully regardless of the indication for hysterectomy.

Our literature review showed that the histopathological classification was endometrioid adenocarcinoma grade 1–2 in 23 of the 25 cases, unknown grade of endometrioid adenocarcinoma in 1 of the 25 cases, and poorly differentiated adenosquamous carcinoma in 1 of the 25 cases. Immunohistochemical (IHC) analysis was performed in 9 of the 24 cases and revealed a typical staining pattern as previously reported [21, 22]. Previous reports have shown that women younger than 45 years rarely developed EC, and the most common subtype of classification in younger women was endometrioid adenocarcinoma grade 1–2 [23, 24]. Although the number was limited, these results suggested that pregnancy did not affect the subtype and IHC staining pattern of EC. We considered that our literature review might be biased because we could include only published literature and cases that made it to the scientific publication stage and this condition might be under-reported; thus, this is the limitation of our study.

The case we presented is rare, and this report highlights several interesting points, as follows: 1. It describes the histopathological analysis of EC during pregnancy; 2. It describes a tumor involving the lower uterine segment and simultaneously the uterine fundus; and 3. It describes the MRI appearance of EC during pregnancy.

Histopathological examination of the specimen revealed EC that presented as a well-differentiated adenocarcinoma with a focal cribriform pattern, back-to-back structure, and a papillary area. Although IHC analysis showed positive expression of estrogen and progesterone receptors, our patient did not demonstrate any metastases, and no recurrence was observed 4 years after the cesarean hysterectomy. These features resemble those of typical grade 1 endometrioid adenocarcinoma [25–27]. We concluded that the high-dose estrogen and progesterone condition during pregnancy did not promote progression of the EC. As shown in Table 1, most authors considered that pregnancy did not worsen the prognosis of EC. Further cases are expected to discuss how the pregnancy affects the prognosis of EC. Moreover, the histopathological and IHC findings in our case showed similar pattern to those of typical EC.

**Table 1:** A summary of the literature review findings for endometrioid carcinoma associated with pregnancy

First author	Year (Reference number)	Age (years)	Timing of diagnosis		Outocome	Period after diagnosis	Symptoms	The results of histopathogical examination	Immunohisotocheical staining	Stage	Surgical treatment
Kovács AG	1996 [5]	35	Abortion	NA	NED	1 year	Abnormal genital bleeding	EA grade 1–2	NA	IA	Brachytherapy + TAH + BSO + RT
The authors hypothesized that pregnancy may adversely affect the tumor growth; however, it cannot be proven because of the limited number of cases.											
Kodama J	1997 [6]	30	Postpartum	7 months	DOD	8 months	Abnormal genital bleeding	Poorly differentiated adenosquamous carcinoma	NA	IIIC	C
The authors opined that an immature, progesterone-unresponsive endometrium could be the possible mechanism of allowing endometrial carcinoma to develop in pregnancy.											
Schammel DP	1998 [7]	38	Abortion	9 weeks	NED	58 months	Infertility	EA grade 1	NA	IA	Repeat curettage with progesterone therapy
		41	Abortion	13 weeks	NED	48 months	Abnormal genital bleeding	EA grade 1	NA	IA	TAH + BSO
		29	Abortion	9–10 weeks	NA	NA	None	EA grade 1	NA	IA	NA
		34	Abortion	13 weeks	NED	12 months	Abnormal genital bleeding	EA grade 1	NA	IA	TAH + BSO
		33	Postpartum	During cesarean delivery	NED	57 months	None	EA grade 1	NA	IA	Repeat curettage with progesterone therapy
The authors considered that the fate of the more advanced-stage tumors with deeper myometrial invasion or high-grade cytologic features may be less subject to the protective effects of gestational progesterone.											
Ayhan A	1999 [8]	44	Abortion	5 weeks	NA	NA	Abnormal genital bleeding	EA grade 1	NA	IA	TAH + BSO + LND + OM
The authors cited a previous report which observed that hCG inhibits the DMBA-induced breast carcinogenesis in rats through an insulin-like growth factor-dependent mechanism.											
Foersterling DL	1999 [9]	31	Postpartum	9 weeks	NED	1 year	Abnormal genital bleeding	EA grade 1	NA	IA	TAH + BSO
The authors opined that in pregnancy-associated endometrial carcinoma, part of the lining undergoes gestational change, whereas another part becomes neoplastic. The portion of the endometrium which becomes neoplastic may be sensitive to estrogen, yet unresponsive to progesterone.											
Vaccarello L	1999 [10]	35	Abortion	9 weeks	NED	31 months	Abnormal genital bleeding	EA grade 1	NA	IA	TAH + BSO
		40	Postpartum	4 months	NED	6 years	Abnormal genital bleeding	EA grade 1	NA	IA	TAH + BSO
		32	Postpartum	4 months	NED	3.5 years	Abnormal genital bleeding	EA grade 2	NA	NA	TAH + BSO
They concluded that with concomitant secretory endometrium, the malignant regions must be progesterone refractory.											
Mitsushita J	2000 [11]	28	Postpartum	6 months	NA	NA	Previous history of endometrioid carcinoma	EA grade 1	ER: positive PR: positive	IA	TAH
The authors did not discuss the association between pregnancy and endometrioid carcinoma.											
Ishioka S	2000 [12]	25	Postpartum	14 months	NED	6 months	Abnormal genital bleeding	EA grade 1	ER: positive PR: negative p53: negative	IA	mRH + BSO + LND
The authors concluded that the occurrence of postpartum EC was extremely rare probably due to the anti-tumor effects of progesterone.											
Ichikawa Y	2001 [13]	35	Postpartum	6 months	NED	3.5 years	Lower abdominal pain	EA grade 1	NA	IB	TAH + BSO + LND + OM + Appendectomy
The authors speculated that high progesterone levels during pregnancy may protect against EC.											
Itoh K	2004 [14]	39	Postpartum	6 months	NED	3 years	Abnormal genital bleeding	EA grade 1	ER: negative PR: negative	IB	TAH + BSO + LND
The authors concluded that the anticancer effect of progesterone during pregnancy was in effect in these tumors.											
Hannuna KY	2009 [3]	34	Abortion	12 weeks	NED	18 months	Abortion	EA grade 1–2	ER: positive PR: positive CK7: positive CK20: negative β-hCG: negative E-cadherin: positive EpCAM: positive Placental alkaline phosphatase: positive	IA	D&C
The authors speculated that the presence of EC might have been related to a hypoxic damage of the chorionic villi. It might suggest a causal correlation between endometrial malignancy and spontaneous abortion.											
The authors found that most case reports of first trimester EA are also reported as arising in a focal lesion.											
Terada T	2009 [15]	29	Concurrent endometrial adenocarcinoma and an early pregnancy loss		NA	NA	Abortion	EA grade 2	ER: positive PR: positive p53: positive vimentin: positive CA19–9: focal positive CA125: positive Ki-67: 80% labelling CEA: negative PTEN: negative p16: negative	NA	Repeat curettage without progesterone therapy
The authors considered that EC associated with pregnancy were mostly in stages IA, and were histologically EAs.											
Akil A	2012 [16]	45	Concurrent endometrial adenocarcinoma and an early pregnancy loss		NA	NA	Abortion	EA grade 1	NA	IA	TAH + BSO + LND
The authors concluded that the routine histological examination of the curettage specimens for all first trimester abortions, independent of the age of the patient, should be encouraged.											
Saciragic L	2014 [17]	36	Abortion	8 weeks	NA	NA	Abnormal genital bleeding	EA grade 1	Ki67: positive	IA	TAH + BSO + LND
The authors discussed that in a woman with progesterone-resistant endometrium, development of endometrial carcinoma could be potentiated by the relatively hyperestrogenic environment of early pregnancy and subsequently allowed to proliferate further due to a lack of response to progesterone.											
Bayoglu Tekin Y	2014 [18]	36	Abortion or ectopic pregnancy	NA	NED	1 year	Ectopic pregnancy	EA grade 1	NA	NA	Curettage with progesterone therapy
The authors though that the presence of EC might have been related to the damage of the chorionic villi, suggesting a causal correlation between EC and spontaneous abortions.											
Zhou F	2015 [19]	40	Concurrent endometrial adenocarcinoma and an early pregnancy loss		NA	NA	Abortion	EA grade 1	ER: positive PR: positive p53: negative	NA	Repeat curettage without progesterone therapy
		33	Concurrent endometrial adenocarcinoma and an early pregnancy loss		NA	NA	Abortion	EA grade 1	ER: positive PR: positive p53: negative	NA	Repeat curettage without progesterone therapy
The authors considered that the careful histological examination of the curettage specimens for all first trimester pregnancy losses should be encouraged.											
Rizzuto I	2019 [20]	29	Pregnancy of 7 weeks gestation	NA	NED	8 years	Abnormal genital bleeding	EA	NA	NA	Serial endometrial biopsy with insertion of a Levonorgestrel intrauterine device
Conservative management for EC in young women is possible including a case with an incidental diagnosis in pregnancy.											
Our case	2019	35	Placenta accreta spectrum	Cesarean hysterectomy	NED	4 years	None	EA grade 1	ER: positive PR: positive p53: negative	IA	Cesarean hysterectomy Laparoscopic BSO + LND

List of abbreviations: *BSO* Bilateral salpingo-oophorectomy, *C* Chemotherapy, *CK7* Cytokeratin 7, *CK20* Cytokeratin 20, *CA19–9* Cancer antigen 19–9, *CA125* Cancer antigen 125, *CEA* Carcinoembryonic antigen, *D&C* Dilatation and curettage, *DOD* Dead of disease, *EA* EA, *EC* endometrioid carcinoma, *EpCAM* Epithelial cell adhesion molecule, *ER* Estrogen receptor, *β-hCG* Human chorionic gonadotropin β-subunit, *LND* Lymph node dissection, *mRH* Modified radical hysterectomy, *NA* Not available, *NED* No evidence of disease, *OM* Omentectomy, *PR* Progesterone receptor, *RT* Radiation therapy, *TAH* Transabdominal hysterectomy

The reason for the presentation of separate tumors at the uterine fundus and lower uterine segment is unknown. Histopathological analysis of both tumors showed similar findings; thus, we concluded that the tumor presented as 2 separate growths at the aforementioned sites owing to enlargement of the uterus during pregnancy (although this remains speculative). Other possibilities considered were metastasis or multi-site involvement of EC. Myometrial invasion was insignificant; thus, we excluded metastasis as a possible etiology. The possibility of multi-site involvement of EC is difficult to exclude; however, the estimated frequency of this condition is low. Therefore, we concluded that the tumor separation could be attributed to the uterine enlargement during pregnancy.

MRI scans were retrospectively analyzed after the cesarean hysterectomy. We observed a lesion in the uterine fundus measuring approximately 3 cm in diameter with signal intensity similar to that of the placenta. Notably, this lesion was separate from the placenta. Clinicians must consider the possibility of EC in women with MRI scans showing such lesions during pregnancy.

In conclusion, our findings in this case suggest that careful analysis of MRI findings during pregnancy and gross examination of the resected uterus (in patients undergoing hysterectomy for obstetric complications) are essential, although EC during pregnancy is extremely rare. The literature review suggested that EC associated with pregnancy seemed to have a good prognosis.

## Data Availability

Not applicable.

## Abbreviations

D&C: Dilatation & Curettage
EC: Endometrial carcinoma
IHC: Immunohistochemical
MRI: Magnetic resonance imaging
PAS: Placenta accreta spectrum
